# An integral vision of pain and its persistence: a whole-person, whole-system, salutogenic perspective

**DOI:** 10.3389/fpain.2025.1641571

**Published:** 2025-08-14

**Authors:** Mark I. Johnson

**Affiliations:** Centre for Pain Research, School of Health, Leeds Beckett University, Leeds, United Kingdom

**Keywords:** pain, integral theory, All Quadrants All Levels (AQAL) framework, salutogenic, community, painogenic environment, evolutionary-mismatch, integrative

## Abstract

Persistent pain remains a significant global health challenge, with prevailing biomedical and biopsychosocial models often falling short in capturing its full complexity. These models frequently lack conceptual and contextual coherence, overlooking the deeply subjective, cultural, and systemic dimensions of pain. As a result, care can become fragmented and suboptimal. This perspective article introduces an integral vision of pain, grounded in the All Quadrants, All Levels (AQAL) framework, which offers a multidimensional approach that integrates subjective experience, objective mechanisms, cultural meaning, spiritual perspectives, and systemic structures. The article outlines how a simplified AQAL framework can serve as a heuristic tool to synthesise individual and collective dynamics—including psychological development and socio-environmental conditions—thereby informing a more comprehensive understanding of pain and its persistence. This includes recognising the role of painogenic environments and the impact of evolutionary mismatch in shaping pain experiences. This integral perspective reframes persistent pain within a salutogenic social model of health, adopting a whole-person, whole-system approach that supports the co-creation of compassionate, community-driven, and context-sensitive care. Ultimately, it reconceptualises persistent pain not merely as a disease state or clinical symptom, but as a dynamic, relational, and meaning-laden experience embedded within the evolving journey of life. This integral vision challenges reductionist paradigms, advancing a more coherent, salutogenic, and humanistic model for understanding and addressing persistent pain.

## Introduction

Persistent (chronic) pain is a significant burden to individual well-being, societal health, and the sustainability of healthcare systems (1–3). Despite advances in and widespread availability of biomedical and psychological interventions, persistent pain continues to affect nearly one-third of the global population—a treatment-prevalence paradox (4–6). Pain is a complex and deeply human phenomenon that encompasses hidden subjective inner states, observable physiological and behavioural correlates, and interconnectedness to shared cultural and socio-ecological systems. Pain science is the systematic study of the biological, psychological, and social mechanisms underlying pain experience associated with actual or potential damaged and/or dysfunctional tissue (7). Historically, the focus of pain science on damaged and/or dysfunctional tissue led to biomedical dominance and reduced emphasis on subjective, cultural, and existential dimensions (8–10). The biopsychosocial model of pain redresses the imbalance created by the dominance of the biomedical model.

The term biopsychosocial denotes an interdisciplinary approach to health, illness, and care delivery, grounded in the interplay between biological processes (e.g., genetics, physiology), psychological dimensions (e.g., emotions, cognition, behaviour), and social contexts (e.g., culture, relationships, socioeconomic status). While representing a significant advancement over reductionist biomedical paradigms—which reduce complex human experiences to their simplest biological components—the biopsychosocial model has faced critique for its limited sensitivity to the subjective experience of pain and for the conceptual fragmentation of its biological, psychological, and social domains (11–20). These domains often become disjointed in both theoretical discourse and clinical practice, resulting in fragmented care and a lack of explanatory coherence that is disconnected from the lived reality of *being* human.

This conceptual disjunction is further compounded by the cultural and epistemological underpinnings of the model itself, which originated within a Western, Educated, Industrialized, Rich, and Democratic (WEIRD) cultural worldview (21). The WEIRD worldview tends to privilege individualism, analytical reasoning, mechanistic interpretations, and impersonal prosociality, which emphasizes cooperation among strangers based on abstract norms, institutions, and rule-based fairness rather than close interpersonal ties (22–26). Such orientations may have driven the reductionist biomedical paradigm and marginalization of relational, spiritual, and existential dimensions of pain that are often foregrounded in non-WEIRD cultural contexts (27, 28). In many indigenous and collectivist traditions, pain is not merely a pathological disruption but a meaningful aspect of life's natural rhythm—interwoven with communal, emotional, and spiritual realities (29–32).

The continued influence of biomedical paradigms has contributed to the recognition of persistent (chronic) primary pain as a distinct (pathological) disease entity in the latest revision of the World Health Organization's (WHO) International Classification of Diseases (ICD-11) (33–35). While this framing offers many advantages, it also legitimizes biomedical interventions that have, at least in part, contributed to systemic harms—such as the global opioid crisis (1, 36). Moreover, the empirical foundation of many clinical interventions remains tenuous, with numerous treatments demonstrating limited efficacy in practice—often attributed to a broader crisis in research quality (37–39). Conceptual ambiguities within pain science, including the conflation of nociception with pain and the reliance on speculative constructs further undermine theoretical clarity (40–42).

A growing body of scholarship contends that pain is too idiosyncratic, context-dependent, and meaning-laden to be adequately captured by mechanistic or reductionist models (43–47). Meta-ethnographic perspectives have likened pain to an ecosystem—dynamic, adaptive, and shaped by intersecting biological, psychological, social, and environmental forces (48, 49). The sobriquet of "sticky pain"—a term coined by Borsook et al. (50) to describe persistent, treatment-resistant pain—reflects the failure of certain pain episodes to resolve. As a colloquial term "stickiness" can be used to draw attention to the ecology of pain and factors in contemporary life that may hinder pain resolution (i.e., socio-ecological risk factors).

While the biopsychosocial model nominally incorporates multiple domains, it does not explicitly engage with existential or phenomenological frameworks—those that foreground temporality, meaning, and the lived experience of suffering (11–17). There is increasing recognition that such perspectives are essential for understanding pain not merely as a clinical symptom but as a socially grounded, deeply human condition (45, 46, 51). However, the prevailing structure of professional knowledge production continues to favour specialization over integration, thereby impeding the development of comprehensive models that reflect the full scope of pain as a lived and relational experience.

In response to these limitations, this article proposes a contextual “integral” view of pain and its persistence that includes all dimensions in a unified way. An integral approach weaves together inter- and transdisciplinary knowledge, lived experience, and reflective inquiry to synthesise subjective experiences of pain with its objective mechanisms, all within the broader cultural, systemic, and environmental contexts of contemporary life. It seeks to avoid reductionism, embrace complexity, and honour both the inner life of the person in pain and the scientific understanding of pain processes. An integral approach emphasises the interconnectedness of the individual within their social and environmental context—supporting a whole-person, whole-system, whole-health perspective (52–56).

The aim of this article is to present an integral vision of pain that seeks to unify diverse disciplinary insights, fostering a whole-person, whole-system, and salutogenic perspective on understanding and addressing the persistence—or stickiness—of pain. Building on previous work, this article extends the author's earlier mapping of pain onto a simplified version of Ken Wilber's All Quadrants All Levels (AQAL) framework (57). Rooted in a contemporary, non-religious understanding of spirituality—as meaning, purpose, and connection to self, others, and nature—the integral, AQAL framework enhances the traditional biopsychosocial approach by incorporating subjective experience, objective mechanisms, cultural meaning, and systemic influences. In doing so, it offers a more holistic, context-sensitive, and experientially anchored understanding of pain and its persistence, framed through an evolutionary mismatch, salutogenic, and socially embedded model of health.

This integral perspective is shaped by the author's own positionality as a white, male academic based at a post-1992 UK university, with over four decades of experience in pain research. Drawing on critical realism, positivist science, and reflexive inquiry, this work blends methodological rigour with integral thinking that combines multiple perspectives—including individual and collective, interior and exterior, science and spirituality. It is informed by Integral Theory, biopsychosocial and salutogenic paradigms, and evolutionary psychology. The author remains critically aware of how his social and disciplinary positioning informs his interpretations of the persistence of pain and its cultural significance, acknowledging the influence of both personal and epistemological standpoints in shaping this multidimensional approach.

## Theoretical foundations and integral framework

The understanding of pain has evolved from spiritual and humoral interpretations to Descartes' mechanistic model and the dominance of biomedicine, before being reframed by Melzack and Wall's 1965 Gate Control Theory, which introduced the nervous system's modulatory role (58). This set the stage for Engel's introduction of the biopsychosocial model in 1977 arguing that complex health conditions require considering not only biological factors but also psychological and social influences (59). Following the adoption of the biopsychosocial model (60), its translation into clinical practice has remained partial, with the social dimension frequently marginalised or insufficiently addressed. Advances in neuroscience reframed pain as a complex brain-generated output shaped by context, emotion, and cognition, prompting the emergence of psychologically informed physiotherapy and interdisciplinary pain management. More recently, scholars have advocated for post-biopsychosocial approaches—such as enactive and 5E frameworks—that emphasise the embodied, embedded, enacted, emotive, and extended nature of pain (11, 13). These developments reflect a growing recognition of pain as a dynamic, lived experience shaped by both internal processes and external environments, reinforcing the need for holistic, patient-centred care.

Ken Wilber, a populist American psychologist/philosopher, developed the concept of the *integral mind* as a means of unifying the physical, emotional, mental, and spiritual dimensions of human experience (61, 62). His work is characterized by an effort to bridge longstanding divides between Eastern and Western philosophies, science and spirituality, and individual and collective perspectives. At its core, integral thinking seeks to cultivate a holistic understanding of reality in which diverse perspectives are not merely acknowledged but actively harmonized (61, 63). In practice, the integral mind reflects an individual's capacity to synthesize multiple dimensions of experience into a coherent worldview. This integral orientation aligns particularly well with existential models of pain, which offer a deeply humanistic perspective—one that recognizes both inner subjective experience and objective bodily processes as inseparable from the shared cultural, environmental, and spiritual dimensions of contemporary life.

Wilber developed the AQAL framework to create a comprehensive model for understanding complex human experiences (61, 64). All Quadrants (AQ) refers to four different perspectives on experience:

• individual-interior [upper-left (UL)]: intrasubjective, personal, inner experience
• individual-exterior [upper-right (UR)]: intraobjective, biology, behavior
• collective-interior [lower-left (LL)]: intersubjective, societal culture and meanings
• collective-exterior [lower-right (LR)]: interobjective, societal systems and structures.

All Levels (AL) refers to stages of development (complexity) that shape how individuals and collectives interpret and respond to an experience over time—represented by levels (stages) of psychological development in the left side “interior” quadrants.

The AQAL framework is underpinned by several core principles that render it particularly suitable for advancing an integral perspective of pain:

• Non-Exclusion: All perspectives—psychological, biological, social, environmental, and spiritual—are considered valid and necessary for a comprehensive understanding.
• Enfoldment: Recognizes that experiences and interpretations evolve across nested levels of personal and cultural development.
• Enactment: Emphasizes that reality is co-constructed through interaction; pain is not merely a passive experience but is shaped by relationships, environments, and meaning-making processes.

These principles resonate strongly with phenomenological and socio-ecological understandings, which emphasize the meaning-laden nature of embodied, embedded and enacted pain experience of people interconnected with the shared culture of others and environmental settings [e.g., (11, 65)]. The AQAL framework's emphasis on the “inner-world” (interiority) and developmental levels of psychological complexity supports the integration of such insights into both clinical and theoretical models (61, 64).

While the biopsychosocial model acknowledges multiple domains, it lacks the structural coherence and contextual depth. In contrast, AQAL integrates:

• Subjective experience and existential meaning of the individual (UL)
• Objective bodily correlates of the individual (UR)
• Cultural narratives and meanings of the collective (LL)
• Systemic and institutional influences of the collective (LR)
• The evolving nature of psychological understandings (levels of development)

Furthermore, the AQAL framework supports a transdisciplinary synthesis by bridging insights from philosophy, psychology, medicine, sociology, ecology, and spirituality. This consolidative capacity makes it an ideal foundation for a comprehensive, whole-person, whole-system, whole-health model of pain and its persistence that is both scientific and existentially meaningful. In this context, the act of modelling becomes inseparable from the phenomenon being modelled. Any framework used to conceptualise pain not only reflects but also shapes how pain is understood, treated, and even experienced. In this sense, models of pain do not merely describe reality—they actively participate in constructing it.

This dynamic is evident in the evolution of pain models themselves. The biomedical [“pain pathway”(*sic*)] model, with its emphasis on tissue damage and pathology, historically shaped clinical practice around physical (biomechanistic) interventions. Nowadays, the biopsychosocial model, incorporating psychological and social dimensions, shapes multidimensional understandings and multidisciplinary clinical practices adopting *patient*-centred approaches. Building on this progression, an integral model—framed through an AQAL, evolutionary-mismatch and salutogenesis (origins of health) lens—seeks to hold multiple perspectives simultaneously to construct *person*-centred, context-sensitive, meaning-making approaches to empower people to actively reshape their living experiences of pain in the modern world. Thus, the vision for an integral framework is not only to represent the persistence of pain more comprehensively, but to actively reshape how it is lived and addressed—expanding the possibilities for healing.

## Mapping pain to the AQAL framework

**Figure 1:**
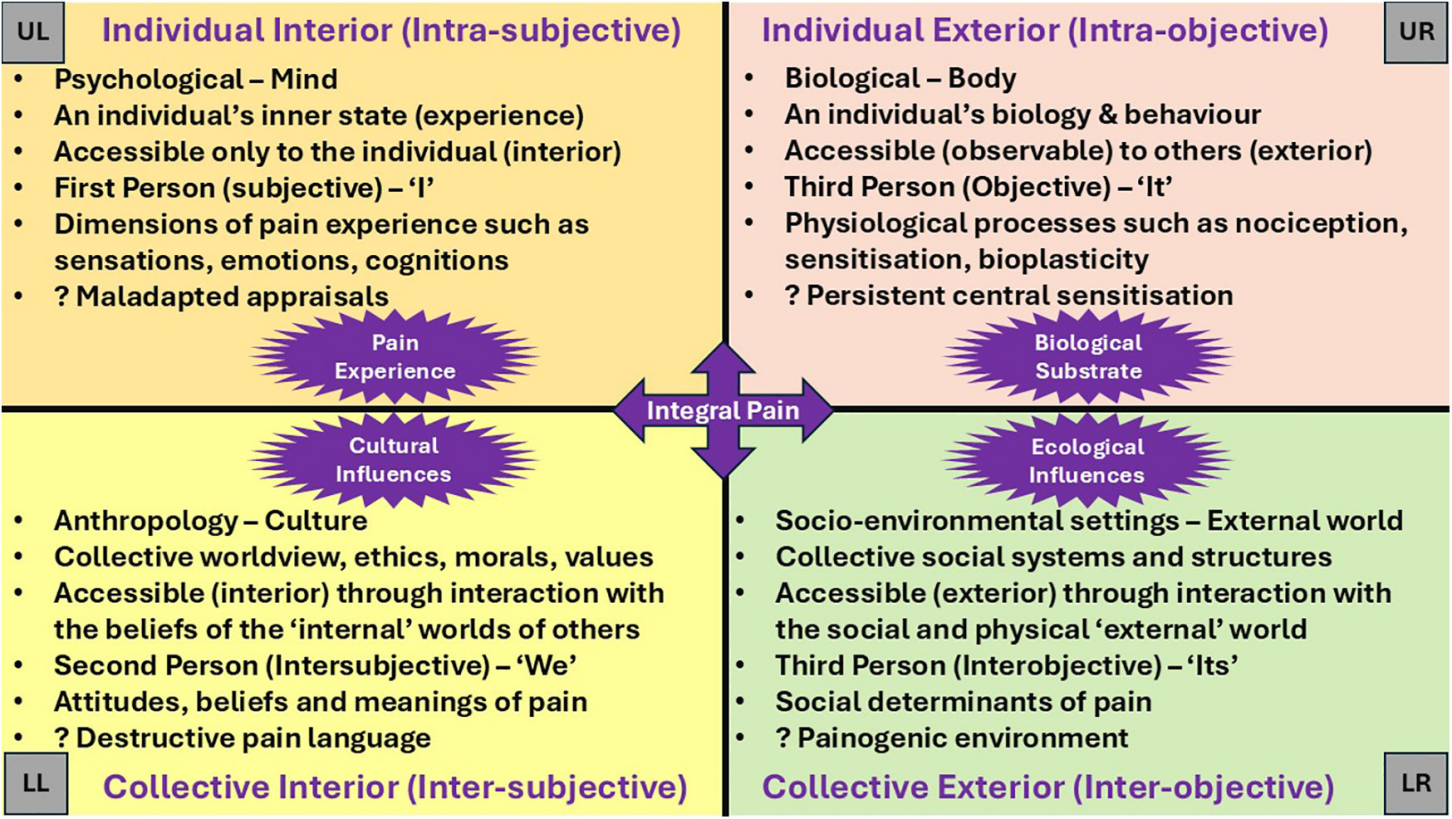
The quadrants framework offers a comprehensive lens through which to understand the multidimensional nature of pain. The Upper Right (UR) quadrant represents the objective, observable aspects of the individual, including neuroscientific and biological mechanisms that govern bodily functions and behaviours. In contrast, the Upper Left (UL) quadrant addresses the subjective, psychological, and experiential dimensions of the individual's inner life. The Lower Left (LL) quadrant encompasses the intersubjective domain, highlighting the cultural, moral, and relational influences that shape meaning within collective society. Finally, the Lower Right (LR) quadrant captures the interobjective dimension, focusing on systemic, structural, and environmental factors that influence the broader socio-ecological context. Together, these quadrants provide a holistic framework for understanding pain as a complex interplay of individual and collective, internal and external factors. Based on (57).

Previously, the author mapped features of pain onto a simplified version of the AQAL framework highlighting certain limitations of the biopsychosocial model while preserving its foundational insights, thereby enabling an extension of the biopsychosocial model's conceptual scope (57). As a heurist tool the simplified AQAL framework is capable of accommodating both personal experience and systemic context in an organizing structure that visualises the location of multidimension aspects of pain, as represented by the four quadrants in Figure 1.

### All quadrants

While the biopsychosocial model emphasizes the interplay of biological, psychological, and social factors at the level of the individual, the simplified AQAL framework broadens this scope by integrating individual, collective, interior and exterior perspectives offering insights that enables a more nuanced and layered understanding within the context of structures and systems of contemporary living. The quadrant structure aligns closely with the multidimensional nature of pain, contextualizing pain within a biopsychosocial-existential paradigm. Pain emerges from an individual's biological processes (UR) to be experienced by the individual subjectively (UL), shaped by collective cultural and existential narratives (LL), and interaction with collective systems and structures of social and physical environments (LR).

Visually and conceptually distinguishing aspects of pain and its persistence between quadrants enhances the clarity of pain theory in ways that the biopsychosocial model alone does not achieve. By locating pain within the subjective (UL) quadrant, AQ mapping helps to expose conceptual errors, such as the reification of pain (the fallacy of misplaced concreteness)—treating pain as a fixed, object-like “thing”—as well as conflating pain with nociception, a miscommunication that continues to obscure theoretical and clinical clarity (40, 41). Quadrant analysis informed by transdisciplinary knowledge provides opportunities to analyse, evaluate and integrate contemporary pain theories such as the Embodied Predictive Processing Theory of pain experience (66), the 5E Enactive Theory of Pain (11), and the Healthy Settings Model of Pain (67), within the contextual reality of contemporary life.

For example, by embedding Bayesian and Active Inference models within a broader integral framework, we gain a more nuanced understanding of how pain is continually remodelled through both internal cognitive processes and external sociocultural structures. Active Inference and Bayesian approaches to cognition offer insights into how pain experience arises from neural tissue. Grounded in the predictive processing paradigm, these models propose that the brain is not a passive recipient of sensory input but an active constructor of experience—constantly generating predictions about the world and updating them in response to sensory evidence in order to minimise prediction error.

In the context of pain, this suggests that the experience of pain is not a direct reflection of tissue damage, but a probabilistic inference shaped by prior beliefs, contextual cues, and learned expectations. Persistent pain may reflect a state in which entrenched priors, encoded within cortical and subcortical networks, infer non-noxious stimuli as threatening or harmful—even in the absence of nociceptive input—thereby sustaining maladaptive predictions that resist revision. Active Inference extends this view by highlighting the role of action in reducing prediction error: neuroplasticity updates entrenched priors encoded in neural networks by acting on the world—or the body—to align sensory input with expectations. This dynamic interplay between perception, cognition, and action underscores how pain is not merely passively experienced but actively constructed and sustained.

A four-quadrant framework provides a structured lens through which this process can be understood. For example, the intrasubjective quadrant (UL) captures the influence of prior beliefs and expectations on a person's inner living experience; the intraobjective quadrant (UR) addresses the physiological and neurological mechanisms underpinning predictive processing; the intersubjective quadrant (LL) reflects the role of cultural narratives and shared meanings in shaping understandings of pain; and the interobjective quadrant (LR) encompasses the systemic and institutional structures that embed and reinforce these predictive models. This integrative perspective not only deepens theoretical insight but also opens new pathways for therapeutic intervention—targeting maladaptive predictions, enhancing agency, and reshaping the experiential reality of pain. Beyond informing intervention, the quadrant framework can also serve as an analytical tool to analyse and evaluate dimensions of existing treatments and practices.

Quadrant mapping reveals the individualistic, downstream bias of current thinking dominated by biomedical and psychological treatment, such as surgery, pharmacotherapy, and psychologically orientated interventions that focus on upper quadrant domains of the individual. Quadrant mapping fronts the collective cultural and socio-ecological dimensions, highlighting their interplay with the evolutionary mismatch between the UR and LR quadrants—namely, the tension between our Paleolithic physiology (UR) and a contemporary physical and social environment (LR) with painogenic influences that prime development and hinder resolution of pain i.e., make pain "sticky" (68, 69). Embedded within the lower quadrants are large-scale social, cultural, economic, and political forces that subtly, and often invisibly, shape individual and communal experiences in harmful or oppressive ways. Some of these influences are insidious such as medicalization of suffering, neoliberal ideologies, capitalist productivity culture, technocratic governance, colonial legacies, structural racism, patriarchy, ableism and, importantly, damage-laden and militaristic medical language (70–74). Some insidious macro-level forces go unrecognized within the conventional biopsychosocial model of pain, including damage-loaded warmongering pain language (70).

### All levels

The AQAL framework also incorporates levels of psychological development across multiple lines of intelligence—cognitive, emotional, moral, and spiritual (61, 64). Levels of development reflect how individuals and societies evolve in their understanding and response to complex phenomena such as pain. They illuminate how beliefs, attitudes, and values toward pain shift over time— for instance, from premodern undifferentiated blends of philosophical, religious, and medical thought; to modernist biomechanistic models, and more recently, to postmodern biopsychosocial perspectives. Psychological developmental sensitivity offers a nuanced understanding of how pain is conceptualized and managed across diverse personal and cultural contexts. Viewing pain through a developmental lens can help explain variations in lived experiences and treatment responses, depending on whether individuals, practitioners, or healthcare systems interpret pain through biomedical, biopsychosocial, holistic, or spiritual frameworks. See reference (57) for further detail on developmental levels in the context of pain, including examples of how understandings of pain evolve across different stages of psychological development—in patients, practitioners, and healthcare systems.

In summary, the AQAL framework offers a more dynamic and personalized understanding of the persistence of pain, including the role of upstream influences—such as cultural narratives, environmental stressors, and systemic inequities—that contribute to painogenic conditions. Hence, an integral worldview would not only consider biopsychosocial domains focussing on the individual but extends this to cultural narratives, social structures and systems, and existential meaning, aiming for a whole-person, whole-system, whole-health understanding.

## Main perspectives

### An integral vision of pain and its persistence

**Figure 2:**
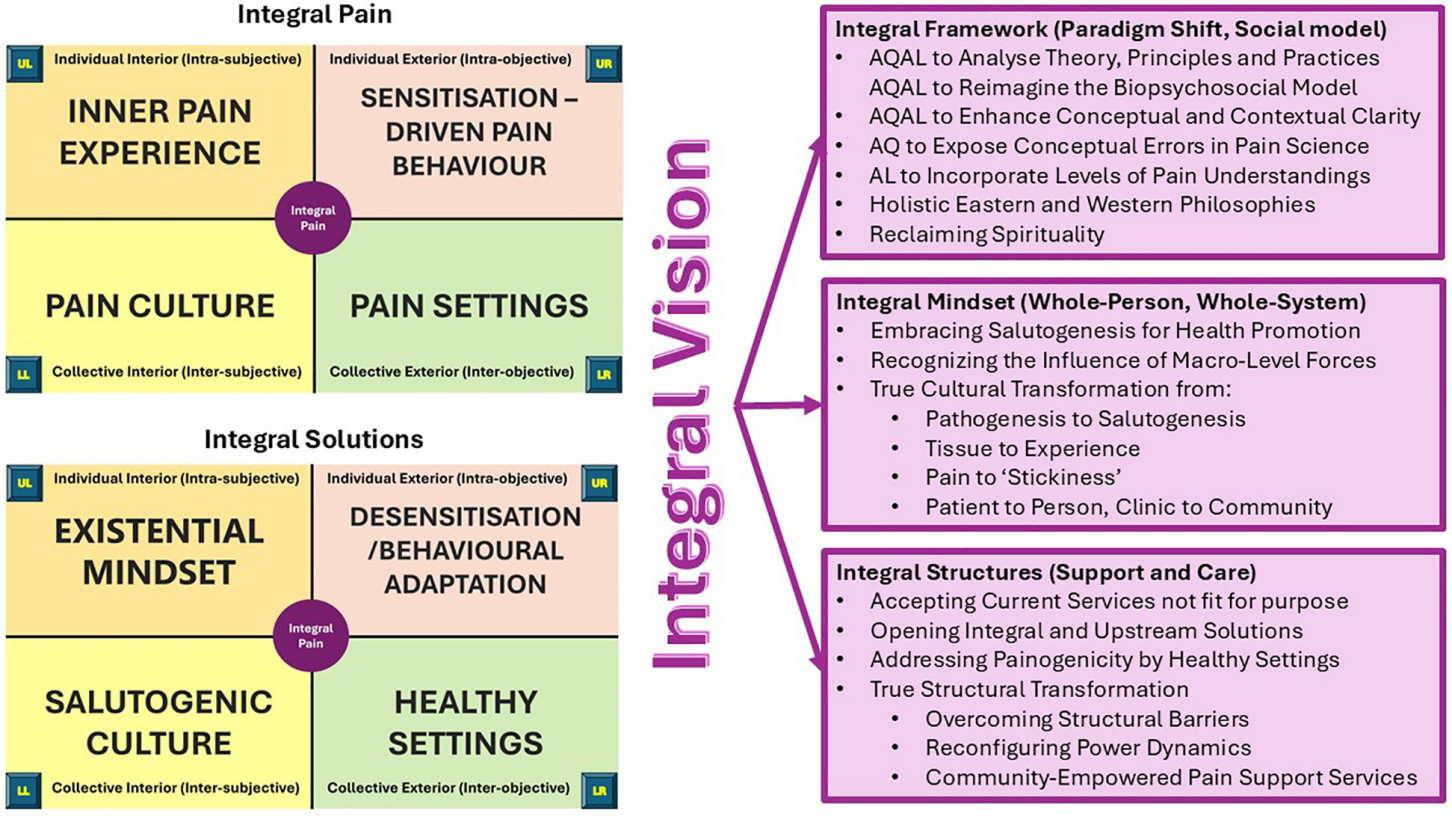
Mapping pain through All Quadrants to inform an integral vision for pain and its persistence. This figure synthesizes the outcome of applying the All Quadrants framework to the experience of pain, revealing a comprehensive, multi-dimensional perspective that transcends reductionist models. By situating pain within all four quadrants—biological, psychological, cultural, and systemic—the mapping exposes conceptual limitations in conventional pain science and extends the biopsychosocial model toward a more integrative, coherent, and holistic paradigm. AQAL framework mapping supports enhanced conceptual clarity, traces the evolution of pain theories, and aligns with holistic philosophies that reclaim spirituality and promote salutogenesis. It highlights the influence of macro-level forces, structural barriers, and power dynamics, while advocating for upstream, community-empowered, and whole-person approaches. This integral mindset reframes pain from a purely clinical issue to a lived, contextual experience—shifting the focus from tissue to experience, from pain to "stickiness," and from pathogenesis to salutogenesis. Ultimately, it lays the foundation for transformative, culturally responsive, and systemically informed pain support services. Based on (57).

The goal of applying the AQAL framework to pain begins with an integral vision: a guiding worldview that seeks to advance how pain and its persistence (stickiness) is understood and engaged with—moving beyond a fragmented, decontextualised and symptom-focused biopsychosocial interpretation toward a more compassionate, whole-person, and context-sensitive integral perspective (Figure 2).

An integral vision of pain reconceptualizes pain as a dynamic, relational phenomenon shaped by subjective experience and meaning-making, biological processes, and interconnectedness with cultural narratives, systems and structures. In doing so, it moves beyond the traditional biopsychosocial model by expanding both its conceptual and contextual scope. Conceptually, it incorporates four irreducible dimensions of human experience: the individual-interior (subjective experience), individual-exterior (biological processes and behaviours), collective-interior (cultural meanings), and collective-exterior (social and environmental settings). It also integrates developmental levels, acknowledging that individuals and systems evolve over time. Contextually, it situates pain within broader ecological, relational, and historical frameworks, highlighting the importance of meaning-making processes that may be overlooked in conventional biopsychosocial approaches [see (57)].

An integral vision of pain reclaims spirituality as a vital dimension of psychological growth and a deepened sense of interconnectedness with broader existential realities, recognising its role in identity, resilience and healing. In a contemporary context, spirituality is understood in non-religious terms—as meaning, purpose, and connection to self, others, and nature (75). Spirituality remains central to end-of-life and palliative care yet is largely absent from guidelines about managing persistent pain (76). This omission reflects a broader tendency to overlook the existential and cultural dimensions of pain in chronic care contexts.

Systematically mapping the multifaceted nature of pain onto a simplified AQAL framework helps illuminate these neglected domains—particularly the collective cultural and socio-ecological factors implicated in an evolutionary mismatch. Evolutionarily conserved physiological systems, shaped by ancestral Paleolithic environments, may be poorly adapted to contemporary “painogenic” conditions—such as sedentary lifestyles, social disconnection, and fragmented healthcare (68). This mismatch may be particularly relevant to the development and persistence of chronic primary pain (69, 77).

An integral vision builds on insights from evolutionary-mismatch (68), salutogenic theory (78, 79), trauma-informed care (80, 81), and a socio-ecological "settings" model (67) to deepen understanding of the persistence—or “stickiness"—of pain. It provides a foundation for restoring coherence across the AQAL quadrants through narrative integration, socially responsive care, and meaning-making practices that support healing as part of an evolving life journey. This approach addresses the evolutionary, environmental, and developmental factors that may contribute to the entrenchment of suffering, aiming not only to account for the persistence of pain but also to inform how it might be more effectively understood, lived with, and addressed. By drawing together diverse perspectives, the integral model offers a framework that supports personal narratives, reduces stigma, and encourages more empathetic, person-centred clinical dialogue.

The integration of salutogenesis—anchored in the principles of comprehensibility, manageability, and meaningfulness—transforms pain from a chaotic disruption into a coherent and meaningful aspect of one's evolving identity. This process, supported by the intrasubjective (UL) and intersubjective (LL) quadrants of the integral model, fosters a growth-oriented, life-affirming approach to pain and associated suffering. By promoting a more coherent and resilient orientation toward healing, salutogenesis also facilitates shared decision-making, affirms lived experience, and strengthens both therapeutic relationships and broader public engagement.

While building on the foundations of the biopsychosocial model, this integral approach seeks to extend its reach by more fully engaging with the lived experience of pain. Through fostering personal agency, narrative coherence, and socially responsive care, it offers a more holistic and potentially empowering path to healing—one that considers not only symptoms, but the broader context of the person's life. In this light, an integral vision invites a gradual cultural shift in how pain and its persistence are conceptualised and addressed, with an emphasis on complexity, compassion, and collective wellbeing.

### An integral approach to healthcare

An integral vision advances the biopsychosocial model by reframing it as a dynamic, context-sensitive framework capable of more fully engaging with the complexity of lived experience, cultural meaning, and structural context in the understanding and treatment of persistent pain. It offers a conceptual architecture for reconfiguring the limits of healthcare, enabling a more inclusive, multidimensional approach that aligns healthcare with the full spectrum of human experience and systemic complexity—supported by communities committed to whole-person, whole-system care.

#### Applying the integral perspective: a case illustration

The case of a 45-year-old office worker with chronic low back pain illustrates how the integral framework can enrich understanding: in the intrasubjective quadrant (UL), they contend with fear of movement, emotional distress, and limiting beliefs such as “I’m broken,” which shape their inner world and influence their pain experience. The intraobjective quadrant (UR) quadrant captures observable physical symptoms including muscle tension, altered posture, and reduced mobility, alongside diagnostic findings from laboratory tests, imaging, or clinical assessments. In the intersubjective quadrant (LL), cultural norms—such as a damage-loaded pain narrative, ideals of masculinity, expectations of productivity, and beliefs about aging—inform how the individual interprets and communicates their pain, while the presence or absence of social support from family, colleagues, or healthcare providers significantly affects their coping capacity. The interobjective (LR) quadrant reflects broader systemic and environmental influences, including living conditions, income, employment status, workplace ergonomics, access to healthcare, and policy frameworks that shape the trajectory of recovery. Together, these dimensions create a whole-person, whole system understanding of the person's situation.

The four-quadrant framework informs a comprehensive care strategy to address the full spectrum of the individual's experience. For example, psychological support such as Cognitive Behavioural Therapy (CBT) or Acceptance and Commitment Therapy (ACT) targets maladaptive beliefs, emotional distress, and fear-avoidance behaviours, fostering greater self-efficacy and meaning-making (UL). Physical rehabilitation, including physiotherapy, graded activity, and movement-based interventions targets observable impairments such as muscle tension, reduced mobility, and postural dysfunction, alongside ongoing review of diagnostic findings (UR). Culturally sensitive pain education and therapeutic dialogue help reframe internalised narratives around pain, masculinity, and productivity, while peer support and family involvement strengthen social cohesion and emotional resilience (LL). Systemic and environmental interventions may include workplace adjustments, improved ergonomics, coordinated multidisciplinary care, and navigation of healthcare access pathways (LR).

Together, these quadrant-aligned strategies advance the biopsychosocial model into a more integral, whole-person, whole-system paradigm—one that supports healing through coherence, connection, and contextual responsiveness across all domains of human experience. Framing this support within a salutogenic perspective, further enhances care by fostering comprehensibility, manageability, and meaningfulness—three foundational elements that help individuals make sense of their pain, feel equipped to navigate it, and find purpose in their recovery journey.

#### Reimagining pain within a salutogenic social model of health

An integral vision supports a reconfiguration of persistent pain within a social model of health with equal partnerships between pain livers, the voluntary, community and social enterprise (VCSE) sector, and healthcare providers. It opens opportunities to frame persistent pain not merely as a clinical symptom but as a socially embedded, meaning-laden phenomenon. It has potential to facilitate a shift from a fragmented, pathogenic model of care to a coherent, salutogenic, and humanistic paradigm. An integral vision supports the co-production of culturally relevant services, tailored to local needs and grounded in shared values. It emphasizes relationality, empowerment, and systemic awareness.

First-person research suggests that individuals living with persistent (chronic) pain benefit from approaches that validate their pain through meaningful explanations so that they feel their personal stories are heard and acknowledged (49, 82). A healing journey with chronic pain involves being reconnected with a sense of identity and purpose to restore self-worth and reintegration into social life—especially within safe and supportive environments that foster a sense of belonging and community (49). Healthcare services often fail to address these experiential and existential needs, focusing instead on downstream interventions. An integral model of care shifts the focus upstream, reimagining support services aligned with health promotion, community-based settings and salutogenic principles.

Antonovsky's concept of *salutogenesis*, introduced in 1979, shifts the focus from the origins of disease to the origins of health (78, 83). Within this framework, the experience of persistent pain is understood not solely as a pathological condition but as an opportunity for individuals to derive meaning and coherence from suffering. Central to this approach is the *sense of coherence*, which enables individuals—either independently or collectively—to interpret pain in a way that supports psychological resilience and positive adaptation. Salutogenesis acknowledges the influence of socio-economic, environmental, and contextual factors on health outcomes, recognising that individuals can maintain or regain health despite ongoing pain.

The salutogenic model of health promotion (78, 84, 85), which emphasizes the origins of health and well-being, aligns closely with the integral approach by highlighting three key salutogenic dimensions:

• Comprehensibility – making sense of pain and its persistence
• Manageability – having the resources to cope with and transform through painful experiences
• Meaningfulness – finding purpose in experiences of pain and its persistence

Salutogenic strategies promote positive health behaviours through education and literacy, social support, and community engagement that validates pain through compassionate listening, self-kindness, and safe social reconnection—principles that are often absent in conventional pain care.

#### Cultural transformation: from integrative to integral

The greatest challenge to integral transformation lies in dismantling entrenched structural hierarchies. Despite widespread acknowledgment of the importance of environmental determinants of health—such as housing, transport, and living conditions—public health and health promotion in countries like the UK, US, and Australia continues to prioritise individual behaviour change. This disconnect is attributed to “lifestyle drift,” where policies initially aimed at addressing upstream social determinants shift focus to downstream lifestyle interventions. Lifestyle drift is partly due to the relative ease of designing and evaluating individual-level interventions compared to structural ones.

In pain management, this trend is particularly evident, with care guidelines remaining individual-centred. Green et al. (65) argue that while health promotion should target structural factors, it is often reduced to personal responsibility, and then medicalised—e.g., lifestyle medicine, prescriptive exercise. Nonetheless, there is growing recognition that health is shaped largely by factors beyond individual control, prompting international calls—such as the Shanghai Declaration (86)—to prioritise supportive environments that make healthy choices more accessible.

Despite long-standing policy commitments to community-empowered care, power, funding, and decision-making remain disproportionately concentrated in healthcare-led institutions, marginalising the very social, relational, and community assets essential for whole-system change. Contemporary healthcare systems promote integrated or integrative pain care, often through multidisciplinary teams that combine biopsychosocial approaches. The International Association for the Study of Pain (IASP) defines integrative care as a coordinated, evidence-based model that blends conventional and complementary treatments (87–89). Integrative services frequently remain extensions of existing systems, relying on mechanism-guided, single-treatment interventions that privilege UR quadrant domains. This narrow focus not only overlooks the socio-ecological complexity inherent in the experience of pain, but also externalises the financial, logistical, and administrative burdens of coordinating multiple services—consequences that often manifest as systemic inefficiencies, increased clinician burnout, and the perpetuation of fragmented care pathways (90–92). A persistent “silo effect” and misalignment between clinicians and patients further hinder collaboration and shared understanding.

Gaudet (56) argues that incremental improvements in existing services are no longer sufficient, calling for systemic “true” transformation across healthcare, culture, and community.

“We are starting from the wrong place. To change the outcomes of the system, we need to change the conversation. We need to start with discovering what gives each of us a sense of meaning and purpose. What matters most deeply in our lives?” (56) p.2

An integral vision is for “true cultural transformation” through reinterpretation of the persistence of pain within a social model of health aligned with whole-person perspectives (93, 94), person-centred health care (51), and unitary care science (95). An integral vision would be to reconfigure pain from always being a pathologic problem to be eliminated, to a meaning-making, growth-orientated experience that warrants interpretation within an evolving life-journey. Such a shift necessitates aligning what is important to patients with reimagined care systems, professional roles, and cultural narratives.

#### Rethinking Pain: community-based support for chronic pain

Overcoming structural and cultural barriers is a major challenge to integral transformation. UK health policy has long advocated for community-empowered care yet implementation has been limited (96, 97). Funding and authority in the UK and elsewhere remain concentrated in healthcare-led services, often sidelining social care, patients and community organizations (98–100). An integral vision of pain challenges this imbalance, advocating for the redistribution of power and the collaborative design of services that reflect the lived realities of those they aim to support. This is exemplified by initiatives such as the UK's Rethinking Pain programme (101, 102) commissioned by a National Health Service (NHS) Integrated Care Board (ICB) and led by a VCSE-sector organisation [Keighley Healthy Living (103)].

Rethinking Pain is a co-produced, community-based pain support service that values relationality, community empowerment, and systemic awareness, developed under NHS clinical governance to address chronic pain and health inequalities through culturally adapted, holistic care. Informed by guidelines for assessing and managing persistent pain published by the National Institute for Health and Care Excellence (NICE) (104) and the Scottish Intercollegiate Guidelines Network (SIGN) (105), the Rethinking Pain service integrates physical, mental, social, environmental, and spiritual dimensions of health. With input from service users, VCSE partners, clinicians, health promoters, and academics, Rethinking Pain offers a three-tier care pathway to support people living with persistent pain through a holistic, culturally responsive approach. The service includes workshops that cover a wide range of topics such as beliefs, faith and spirituality, “your story”, creative therapies, emotional wellbeing, keeping active, diet therapy, sleep therapy, and pain education. These workshops are delivered in various languages to ensure they are accessible and culturally relevant. Service users also engage in personalised care planning, working alongside trained health coaches to co-develop strategies tailored to their individual physical, emotional, social and spiritual needs (102). Grounded in salutogenic principles and community ownership—with health coaches as the cornerstone of delivery—Rethinking Pain enhances engagement, treatment adherence, and equity by aligning care with diverse cultural values and lived experiences. While not explicitly structured around the AQAL framework, the Rethinking Pain service reflects many of its core principles, particularly its commitment to whole-person, whole-system, whole-health care for individuals with persistent pain not linked to serious underlying medical conditions.

## Challenges and priorities

Looking ahead, the AQAL framework offers a powerful tool for enhancing service design and delivery by ensuring that all fundamental dimensions of the pain experience—individual and collective, internal and external—are systematically addressed. Its integrative architecture promotes conceptual and contextual coherence, helping services avoid reductionist tendencies and instead embrace the full complexity of human experience. By accommodating different levels of psychological development and cultural worldviews, AQAL can guide the tailoring of interventions and narratives to meet the evolving needs of diverse individuals and communities. This makes it especially valuable for designing dynamic, community-based, person-centred care models that are both inclusive and adaptive—advancing the biopsychosocial model into a truly integral paradigm.

Despite its conceptual strengths, the AQAL framework has had limited exposure in peer-review academic literature and lacks empirical validation. While theoretically rich, levels of psychological development as formulated by Wilber (61, 62, 64, 106) are conceptually fluid and may be prone to overinterpretation, e.g., transpersonal levels of development lack mainstream psychological acceptance (107, 108).

A common critique of Wilber's integral framework is its perceived complexity, which not only encompasses quadrants and levels but also includes lines of development, states of consciousness, and types—making it challenging to apply in practical or clinical contexts without significant interpretive effort (63, 107, 108). A challenge in applying the integral model to clinical practice lies in the overwhelming complexity that emerges when attempting to account for every possible variable. This level of detail introduces a substantial computational burden, making real-world implementation difficult without deliberate simplification. To address this, model reduction becomes essential—a process of selectively narrowing the scope to focus on the most relevant and influential variables. Achieving model reduction within an integral vision of pain, while preserving its theoretical depth and multidimensional scope, requires balancing conceptual richness with clinical usability. This can be accomplished by adopting the four quadrants as the primary heuristic framework and selectively incorporating developmental levels to align narratives with an individual's worldview, thereby simplifying the model into a more accessible and clinically useful tool (57).

Rather than aiming for exhaustive quadrant coverage, clinicians can use the quadrants as a reflective guide to identify patterns and gaps in care. Translating the model into practical tools for practitioners and livers—such as structured checklists or narrative-based guides—would further enhance applicability. Distributing quadrant responsibilities across interdisciplinary teams fosters shared ownership and would reduce cognitive burden, enabling the AQAL framework to function as a flexible, coherent, unifying lens. When integrated with established models such as the biopsychosocial framework or Bayesian and Active Inference theories, AQAL supports enhanced clinical reasoning without overwhelming practitioners.

To enhance the applicability and scholarly credibility of the AQAL framework within pain science, several research priorities warrant attention. Cultural and contextual analyses are needed to examine how sociocultural and environmental factors contribute to the persistence of pain and influence treatment outcomes. At the macro level, it is important to investigate the impact of broader social forces—such as public policy, socioeconomic inequality, and media representations—on the lived experience of pain. Evaluative studies of AQAL-informed policies and community-based programs can help assess their effectiveness in improving health outcomes and promoting health equity. Integrating diverse research paradigms may also facilitate a more holistic understanding of pain, shifting the focus beyond symptom alleviation to encompass broader outcomes such as wellbeing and quality of life. Interdisciplinary collaboration across all quadrants—biological, psychological, cultural, and socio-ecological—is essential to ensure comprehensive analysis. Engaging a wide range of stakeholders, particularly individuals with lived experience of persistent pain, is vital for validating research findings and ensuring their relevance. Finally, the design of interventions should remain adaptive, incorporating ongoing stakeholder feedback and responding to evolving cultural narratives.

## Conclusion

This article proposes that an integral perspective—drawing on the AQAL framework, evolutionary mismatch theory, and salutogenic principles—offers a novel way to reconceptualize the nature and treatment of persistent or "sticky" pain. From this foundation emerges an integral model, a practical tool that maps the biological, psychological, social, cultural, and existential dimensions of pain across individual and collective contexts, offering a more coherent and holistic approach that acknowledges pain as a deeply embedded, meaning-rich experience.

By conceptualising pain as both a physiological event and a meaning-laden experience, this approach interprets its persistence as a reflection of broader cultural and environmental conditions in contemporary life. It challenges the limitations of reductionist paradigms and fragmented understandings by reframing the biopsychosocial model to account for the dislocated domains of pain—where biological, psychological and social influences are often treated in isolation rather than as interconnected elements. This reconceptualization positions pain as a dynamic, relational, and context-sensitive phenomenon, capable of more fully engaging with the complexity of lived experience, cultural meaning, and modern habitats.

By enhancing conceptual clarity, the AQAL framework invites the general public into a more nuanced and relatable understanding of pain by building on established pain education concepts—such as an “overprotective brain *(sic)*” and reductive explanations focussing solely on a hyperexcitable nervous system. Through narrative and storytelling, mapping lived experiences across four interrelated domains can be brought vividly to life. For instance, a woman living with fibromyalgia may describe how her body flares up under stress (UR), how she feels emotionally dismissed and misunderstood (UL), how her daily environment lacks appropriate adaptations to support her condition (LR), and how her family struggles to believe that her pain is as severe as she reports (LL). These stories help individuals explain their pain within a broader, integrative framework—one that validates their experience, challenges fragmented models of care, and opens new pathways for healing that are both compassionate and contextually grounded through salutogenesis to catalyse a sense of coherence. Such conceptual clarity lays the foundations to develop an integral vision that empowers communities and the VCSE sector services to co-create health-promoting solutions. With the support of healthcare providers, this model exemplifies a shift toward relational, whole-person, whole-system care.

Ultimately, an integral vision calls for a cultural transformation in how persistent pain is conceptualized, communicated, and addressed—emphasizing the importance of meaning-making and ensuring access to resources that support coping and psychological growth through health promotion. An integral vision of pain places individual empowerment at its core—recognizing each person's capacity to transform painful experiences into transformative purpose (109). Realizing this integral vision requires a shift from institutional control to fostering personal agency within empowered communities rooted in the cultural and environmental realities of contemporary life.

## Data Availability

The original contributions presented in the study are included in the article/Supplementary Material, further inquiries can be directed to the corresponding author.
